# Real-World Osilodrostat Effectiveness and Safety in Nonpituitary Cushing Syndrome

**DOI:** 10.1210/clinem/dgaf633

**Published:** 2025-11-19

**Authors:** Antoine Tabarin, Jérôme Bertherat, Bénédicte Decoudier, Hélène Lasolle, Hervé Lefebvre, Delphine Drui, Charly Vaillant, Julia Morera, Frédéric Castinetti, Justine Cristante, Nicolas Chevalier, Sarah Fodil-Cherif, Jacques Young, Andrea Piacentini, Wence Agbotounou, Mario Maldonado, Arnd Mueller, Nicolas Scheyer

**Affiliations:** Department of Endocrinology, Diabetes and Nutrition, Centre de Référence des Maladies Rares de la Surrénale, CHU de Bordeaux, 33604 Pessac, France; Department of Endocrinology, Centre de Référence des Maladies Rares de la Surrénale, Hôpital Cochin, AP-HP, and Université Paris-Cité, 75014 Paris, France; Department of Endocrinology, CHU de Reims, 51100 Reims, France; Department of Endocrinology, Hôpital Louis Pradel, Hospices Civils de Lyon Bron Cedex, Lyon 1 University, 69500 Lyon, France; Université de Rouen Normandie, INSERM, NorDiC UMR 1239, Department of Endocrinology, Diabetes and Metabolic Diseases, CHU Rouen, 76000 Rouen, France; Department of Endocrinology, CHU de Nantes, 44093 Nantes, France; Department of Endocrinology, CH du Mans, 72037 Le Mans, France; Department of Endocrinology, CHU de Caen, 14033 Caen, France; Aix-Marseille University, INSERM, MMG, Department of Endocrinology, La Conception Hospital, Assistance Publique Hopitaux de Marseille, 13005 Marseille, France; Université Grenoble Alpes, Service d’Endocrinologie, CHU Grenoble Alpes, 38043 Grenoble, France; Department of Endocrinology, CHU de Nice, 06003 Nice, France; Department of Endocrinology and Diabetes, CHU de Montpellier, 34295 Montpellier, France; Department of Endocrinology, Assistance Publique Hôpitaux de Paris, Bicêtre Hospital, Paris Saclay University, 94270 Le Kremlin-Bicêtre, France; Recordati SpA, 20148 Milan, Italy; Recordati AG, 4057 Basel, Switzerland; Recordati AG, 4057 Basel, Switzerland; Recordati AG, 4057 Basel, Switzerland; Department of Endocrinology, Diabetes and Nutrition, CHRU de Nancy, Hôpital Brabois et Université de Lorraine, 54500 Vandœuvre-lès-Nancy, France

**Keywords:** Cushing syndrome, osilodrostat, effectiveness, safety, hypercortisolism

## Abstract

**Context:**

Osilodrostat's clinical development program mostly enrolled Cushing disease patients. Data in nonpituitary Cushing syndrome (CS) patients are limited.

**Objective:**

This work aims to evaluate osilodrostat effectiveness and safety in nonpituitary CS in real-world practice in France.

**Methods:**

A retrospective, observational study (LINC 7; NCT05633953) was conducted in a multicenter institutional practice setting. Data for patients who initiated osilodrostat under the French Autorisation Temporaire d’Utilisation program or, once approved, in routine clinical practice were extracted retrospectively for 36 months or less (2019-2022). Participants included 103 adult nonpituitary CS patients: ectopic adrenocorticotropin secretion (EAS), n = 53; adrenocortical carcinoma (ACC), n = 19; adrenal adenoma (AA), n = 17; and bilateral adrenal nodular disease (BND), n = 14. Forty-three patients remained on osilodrostat throughout the observation period. Median (minimum-maximum) osilodrostat exposure and baseline dose were 177 days (1-1178 days) and 5.0 mg/day (1-60 mg/day), respectively. The main outcome measure was the proportion with mean urinary free cortisol (mUFC) less than or equal to the upper limit of normal (ULN) at Wk 12 (modified intention-to-treat [mITT] population: all enrolled patients with ≥12 weeks' follow-up, excluding patients without Wk 12 mUFC for nonsafety reasons).

**Results:**

Osilodrostat was initiated and titrated based on investigator judgment. Cortisol decreased by Wk 4, remaining stable thereafter. Twenty-three of 52 patients (mITT, 44.2%; 95% CI, 30.5%-58.7%) had mUFC less than or equal to the ULN at Wk 12 (missing values reported as nonresponders). Forty-five of 52 had Wk 12 mUFC available; the proportions with mUFC less than or equal to the ULN by etiology were as follows: EAS, n = 12/29 (41%); ACC, n = 4/6; AA, n = 1/3; and BND, n = 6/7. The most common (≥15%) treatment-emergent adverse events were adrenal insufficiency (28%) and hypokalemia (18%). Twenty-nine patients (EAS, n = 24; ACC, n = 5) died from adverse events (n = 1 assessed as osilodrostat related by investigator), most commonly neoplasm progression (n = 11).

**Conclusion:**

Osilodrostat is a suitable treatment for endogenous CS of various nonpituitary etiologies.

Endogenous Cushing syndrome (CS) is a rare disorder caused by excess cortisol, associated with increased morbidity and mortality risk across varying etiologies and severities of hypercortisolism (1, 2). Cushing disease, caused by a pituitary adenoma, comprises most (60%-70%) endogenous CS cases (2). However, patients may present with nonpituitary causes, including ectopic adrenocorticotropin secretion (EAS; 6%-10%), adrenal adenomas (AAs) and carcinomas (20%-30%), and bilateral adrenal nodular disease (BND; 5%), also referred to as bilateral adrenal nodular hyperplasia (1, 2). Patients with nonpituitary CS are a heterogeneous population, having diverse manifestations of hypercortisolism (3), the severity of which varies between etiologies but is usually greater in patients with EAS or adrenocortical carcinoma (ACC) than other etiologies (1, 2, 4-6). The greater severity of hypercortisolism in patients with EAS or ACC than in those with AA or BND is likely to contribute to increased morbidity and mortality (7-9). Moreover, patients with EAS and ACC have underlying neoplasms, which largely contribute to the reduced overall survival rates observed in these patients (7-9).

Surgery for the causal tumor is the first-line treatment for most patients with CS; however, medical therapy represents an important therapeutic option for patients who are temporarily or permanently ineligible for, refuse, or do not respond to surgery (1, 10, 11). Others may benefit from preoperative medical therapy to improve their clinical condition before surgery or as a bridging therapy (12-14). Some patients with CS and severe hypercortisolism urgently require lifesaving treatment to rapidly lower cortisol levels, whereby the dose prescribed by the treating physician can be above the recommended starting dose (4, 10, 15).

Osilodrostat is a potent oral inhibitor of 11β-hydroxylase, which catalyzes the final step of cortisol synthesis. The osilodrostat clinical development program has shown that it provides rapid, sustained cortisol reductions, alongside improvements in cardiovascular and metabolic-related parameters, in patients with Cushing disease (LINC 2, 3, and 4) (16-21) and nonpituitary CS (phase 2 Japanese study) (22). However, data for patients with nonpituitary CS are limited (22). Several case reports and small series provide early insight into real-world osilodrostat use across various nonpituitary CS etiologies (22-25). Osilodrostat initiation and titration in these studies was based on clinical judgment, patient clinical condition, and understanding of osilodrostat pharmacology to achieve treatment goals. To improve understanding of real-world osilodrostat use, LINC 7 (NCT05633953), a retrospective, observational study of osilodrostat in patients with nonpituitary CS, was conducted to evaluate osilodrostat effectiveness and safety in this heterogeneous patient population in real-world settings. See the supplementary appendix (26) for a plain-language summary of the study.

## Materials and Methods

### Patients

Adults in France with an existing CS diagnosis according to current guidelines, excluding Cushing disease, were enrolled in this retrospective, observational study. Patients initiated osilodrostat between implementation of temporary authorization for use in France (Autorisation Temporaire d’Utilisation, April 2019) and commercialization of osilodrostat in France (June 2020), or in routine clinical practice between commercialization of osilodrostat in France (June 2020) and the end of the retrospective follow-up period (December 16, 2022). Identified nonpituitary CS etiologies were EAS, ACC, AA, and BND. Patients were excluded if they had participated in an interventional clinical trial during the study period or had pseudo-, cyclic, or iatrogenic CS. The study was conducted in accordance with the Declaration of Helsinki, with approval of the study protocol by the French Health Data Hub according to local legal requirements; patients provided consent to access their medical records before data collection. The LINC 7 trial is registered at ClinicalTrials.gov (NCT05633953).

### Study Design

LINC 7 was a multicenter, observational, noncomparative, retrospective chart review conducted in France. Data were extracted from patients’ existing medical records collected during the French Autorisation Temporaire d’Utilisation or in routine clinical care. Patient data were extracted retrospectively for up to 36 months.

### Analysis Populations

Data are reported for the following populations: safety: all enrolled patients who received at least one osilodrostat dose between April 2019 and the end of the retrospective follow-up period (December 16, 2022); intention to treat (ITT): all enrolled patients who met all inclusion criteria and received osilodrostat, with 12 weeks or more of follow-up (for the primary analysis, patients who had a missing mean urinary free cortisol [mUFC] value for any reason were classed as nonresponders); modified ITT (mITT): all patients in the ITT population, excluding those who, for any reason except safety, did not have an mUFC measurement at 12 weeks (patients who had missing mUFC values for safety reasons were classed as nonresponders); on-treatment population: all patients taking osilodrostat at each time point analyzed. Selected outcomes were analyzed in patients with EAS, ACC, AA, and BND.

### Assessments and Study End Points

Assessments of mUFC, morning serum cortisol, late-night salivary cortisol (LNSC), and cardiovascular and metabolic-related parameters were performed based on the clinical practice of the treating physician; data were extracted into electronic case-report forms. Each parameter was assessed at osilodrostat initiation and, if data were available, at 4, 8, 12, 18, 24, and 36 weeks and every 12 weeks thereafter until the patient was lost to follow-up, death, end of the observation period, or treatment discontinuation, whichever occurred first. If 2 or more 24-hour UFC measurements were provided at the same time point, mUFC was computed; if only 1 measurement was available, this was considered the mUFC for that visit. mUFC, LNSC, and morning serum cortisol measurements were performed at reference laboratories within university hospitals; normal ranges were provided for each cortisol measurement collected, and cortisol normalization was determined based on these ranges.

The primary end point was the number and proportion of patients with mUFC less than or equal to the upper limit of normal (ULN) at 12 weeks and was analyzed for the ITT and mITT populations. Secondary end points included the proportion of patients with mUFC less than or equal to the ULN over time (on-treatment population); normalization of cortisol measures (mUFC, LNSC, morning serum cortisol, or a composite of the 3 [any cortisol measure (ACM)]) over time; changes from baseline in cortisol and cardiovascular and metabolic-related parameters over time; change from initial dose at available time points; and the proportion of patients with treatment-emergent adverse events (TEAEs; safety population). The end point for analyzing ACM was dependent on the analyte available: mUFC was used regardless of morning serum cortisol and LNSC; if mUFC was not available, LNSC was used; if neither mUFC nor LNSC was available, morning serum cortisol was used. LNSC levels over time are not reported because of small patient numbers during the observation period.

### Statistical Methods

There was no statistical hypothesis testing; power was not formally calculated, and no formal sample-size calculations were performed. The planned sample size of 100 was based on practical considerations. Categorical data are presented by frequencies and percentages; continuous data are summarized by mean (95% CI) or median (minimum-maximum) values. For drug exposure, the date of first dose was recorded; exposure dates were noted only if the dose was changed. If the last dose was changed to 0 mg, the date of this occurrence was considered end of treatment. If the patient was still taking osilodrostat on the last day of observation, that date was recorded as end of treatment. Based on the retrospective nature, missing data were not replaced in the statistical analysis of the primary and secondary end points. Where there were many missing values for a patient, a decision was made following consultation with the marketing authorization holder regarding handling of these data, prior to database lock. In the ITT and mITT population analyses, patients with missing UFC values were recorded as nonresponders. Missing dates were entered where applicable when determining treatment period and/or days since a certain time point, following a conservative approach.

## Results

### Patient Disposition and Baseline Characteristics

**Figure 1.**
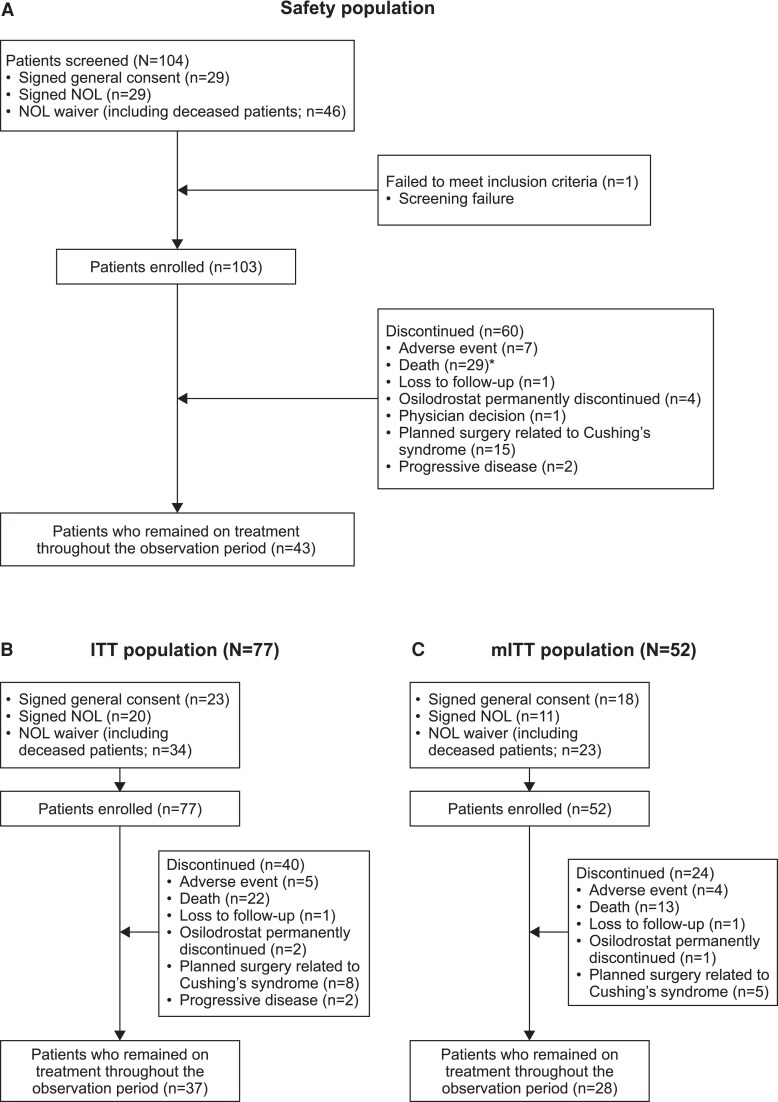
Patient disposition for the A, safety population; B, intention-to-treat (ITT) population; and C, modified ITT (mITT) population. *An additional patient died, and the reason for death is unknown. At the time of database lock, no related serious adverse event record was entered in the electronic data capture database. As such, this patient was not included in the disposition analysis of the safety population. Abbreviation: NOL, no objection letter.

Of 104 consenting patients, 1 was excluded because he had Cushing disease. A total of 103 patients were enrolled in the safety population: EAS, n = 53 (51.5%); ACC, n = 19 (18.4%); AA, n = 17 (16.5%); BND, n = 14 (13.6%). Seventy-seven and 52 patients met the criteria for inclusion in the ITT and mITT populations, respectively. Overall, 43 of 103 (41.7%) patients remained on osilodrostat throughout the observation period, and 60 of 103 (58.3%) discontinued. The reasons for discontinuation were death (n = 30/103, 28.8%), planned surgery for CS (n = 15/103, 14.6%), adverse event (AE; n = 7/103; 6.8%), osilodrostat permanently discontinued (n = 4/103; 3.9%), progressive disease (n = 2/103; 1.9%), loss to follow-up (n = 1/103; 1.0%), and physician decision (n = 1/103; 1.0%; Fig. 1A). Patient disposition for the ITT and mITT populations are presented in Fig. 1B and 1C. Death was the most common reason for discontinuation in patients with EAS (n = 25/53, 47.2%) or ACC (n = 5/19, 26.3%), while in patients with AA or BND, it was planned surgery for CS (n = 4/17, 23.5% and n = 4/14, 28.6%; Supplementary Fig. S1 (26)).

In the safety population, mean (SD) age was 59.3 years (15.5 years); 63 (61.2%) patients were female. Seventy (68.0%) patients received osilodrostat monotherapy: a total of 63 (61.2%) were treatment naive, and 7 (6.8%) received previous medical therapy for CS and switched to osilodrostat. Thirty-three (32.0%) were treated with osilodrostat in combination with another medical therapy for at least 1 day. The medications used in combination with osilodrostat, and their duration of use, are presented in Supplementary Table S1 (26). Overall, mean (SD) baseline mUFC was 20.0 × ULN (45.4 × ULN). Baseline mUFC was highest in patients with EAS and lowest in patients with BND (Table 1). Some patients, mostly those with BND, had mild biochemical intensity of disease at baseline. Overall, 18 patients had an mUFC less than or equal to the ULN at baseline: EAS, n = 6; ACC, n = 1; AA, n = 5; BND, n = 6. Of these 18 patients, 10 had received previous medical therapy for CS: EAS, n = 6; BND, n = 3; AA, n = 1.

**Table 1.** Patient baseline characteristics

	Safety population, N = 103	EAS, n = 53	ACC, n = 19	AA, n = 17	BND, n = 14
Mean age, (SD), y	59.3 (15.5)	61.5 (12.9)	60.9 (18.8)	50.5 (17.9)	59.3 (14.6)
Sex, n (%)					
Female	63 (61.2)	28 (52.8)	11 (57.9)	16 (94.1)	8 (57.1)
Male	40 (38.8)	25 (47.2)	8 (42.1)	1 (5.9)	6 (42.9)
Mean weight, (SD), kg	76.6 (22.3)	74.4 (21.3)	81.0 (23.8)	74.2 (24.8)	80.7 (21.3)
Mean BMI (SD), kg/m^2^	27.8 (6.9)	26.7 (6.5)	28.6 (7.6)	28.0 (7.9)	29.9 (6.1)
Mean mUFC, × ULN (SD)*^a^*	20.0 (45.4)	34.7 (60.9)	8.9 (12.4)	4.8 (7.1)	1.5 (2.3)
Median mUFC, × ULN (min-max)*^a^*	3.4 (0.0-317.4)	7.3 (0.1-317.4)	5.0 (0.0-49.5)	2.0 (0.3-25.4)	0.4 (0.1-7.4)
Type of intervention, n (%)					
Treatment naive	63 (61.2)	28 (52.8)	12 (63.2)	14 (82.4)	9 (64.3)
Switch group*^b^*	7 (6.8)	5 (9.4)	1 (5.3)	1 (5.9)	0 (0.0)
Combination therapy	33 (32.0)	20 (37.7)	6 (31.6)	2 (11.8)	5 (35.7)
Reason for osilodrostat use, n (%)					
Short-term use*^c^*	17 (16.5)	1 (1.9)	7 (36.8)	7 (41.2)	2 (14.3)
Chronic use*^d^*	86 (83.5)	52 (98.1)	12 (63.2)	10 (58.8)	12 (85.7)

Patients in the BND subgroup were described as having adrenal hyperplasia in the electronic case-report form for data collection for this study and the clinical study report.

Abbreviations: AA, adrenal adenoma; ACC, adrenocortical carcinoma; BMI, body mass index; BND, bilateral adrenal nodular disease; CS, Cushing syndrome; EAS, ectopic adrenocorticotropin secretion; max, maximum; min, minimum; mUFC, mean urinary free cortisol; ULN, upper limit of normal.

^*a*^Based on patients with baseline mUFC and ULN values: safety population, n = 73; EAS, n = 36; ACC, n = 15; AA, n = 13; BND, n = 9.

^*b*^Switch group: all patients who were treated with osilodrostat as monotherapy but were taking any medical therapy for CS and discontinued these prior to the start of osilodrostat.

^*c*^Short-term use: patients who took osilodrostat for less than 12 weeks, had or were scheduled for surgery for CS, and were not restarted on osilodrostat immediately after the procedure.

^*d*^Chronic use: all other patients.

Baseline characteristics by treatment population are presented in Supplementary Table S2 (26).

### Osilodrostat Dose and Exposure

Overall, mean (SD) osilodrostat exposure was 37.3 weeks (36.9 weeks). Median (minimum-maximum) osilodrostat exposure and dose are described in Table 2. In patients who received chronic (≥12 weeks) osilodrostat treatment (n = 86), median (minimum-maximum) osilodrostat exposure was 232 days (1-1178 days). See Supplementary Table S3 (26) for a summary of osilodrostat dose changes during the study. Of 103 enrolled patients, 61 received concomitant glucocorticoids; 38 received concomitant glucocorticoids (hydrocortisone) as part of a block-and-replace regimen. The median (minimum-maximum) initial daily osilodrostat dose was 10.0 mg/day (1-60 mg/day) in patients treated according to a block-and-replace regimen and 4.0 mg/day (1-60 mg/day) in patients who were treated with osilodrostat alone. The median (minimum-maximum) average daily dose was 13.8 mg/day (1-60 mg/day) and 7.0 mg/day (0.5-90 mg/day) in each subgroup, respectively.

**Table 2.** Osilodrostat exposure and dose

	Safety population, N = 103	EAS, n = 53	ACC, n = 19	AA, n = 17	BND, n = 14
Median exposure (min-max), d					
	177 (1-1178)	245 (10-846)	47 (14-797)	72 (1-873)	192.5 (28-1178)
Median osilodrostat dose (min-max), mg/d					
Baseline	5.0 (1-60)	7.0 (1-60)	6.0 (4-60)	4.0 (1-20)	4.0 (1-10)
Wk 12	6.0 (0-60)	10.0 (0-60)	30.0 (0-60)	2.5 (0-8)	4.0 (0-20)
Wk 24	10.0 (0-120)	14.0 (0-60)	20.0 (4-120)	4.0 (2-10)	10.0 (1-20)
Wk 36	10.0 (0-120)	16.0 (0-60)	12.0 (7-120)	4.0 (2-8)	7.0 (1-12)

Abbreviations: AA, adrenal adenoma; ACC, adrenocortical carcinoma; BND, bilateral adrenal nodular disease; EAS, ectopic adrenocorticotropin secretion; max, maximum; min, minimum.

### Effectiveness of Osilodrostat

In the mITT population, 23 of 52 (44.2%; 95% CI, 30.5%-58.7%) patients had mUFC less than or equal to the ULN at week 12 (primary end point; 7 patients had missing values and were recorded as nonresponders). In the ITT population (N = 76/77; 1 patient initiated osilodrostat treatment but had <12 weeks’ follow-up at week 12), 23 of 76 (30.3%; 95% CI, 20.2%-41.9%) had mUFC less than or equal to the ULN at week 12 (31 patients had missing values and were recorded as nonresponders). Outcomes for the 45 patients in the mITT population with an available mUFC assessment at week 12 overall, by etiology, baseline mUFC severity, and type of intervention, are presented in Table 3. See the supplementary appendix (26) for sensitivity analyses that were conducted for the primary end point in different patient populations.

**Table 3.** Number of patients with mean urinary free cortisol (mUFC) less than or equal to the upper limit of normal at week 12 based on patients with available mUFC assessments (modified intention-to-treat population)

mITT population subgroup	No. of patients, n/N (%; 95% CI)
Overall	23/45 (51.1; 35.8-66.3)
Etiology of CS	
EAS	12/29 (41.4; 23.5-61.1)
ACC	4/6*^a^*
AA	1/3*^a^*
BND	6/7*^a^*
Baseline mUFC severity*^b^*	
<2 × ULN	10/14 (71.4; 41.9-91.6)
2-5 × ULN	2/8*^a^*
>5 × ULN	7/15 (46.7; 21.3-73.4)
Type of intervention	
Osilodrostat monotherapy	21/37 (56.8; 32.5-63.3)
Osilodrostat combination therapy	2/8*^a^*

Abbreviations: AA, adrenal adenoma; ACC, adrenocortical carcinoma; BND, bilateral adrenal nodular disease; CS, Cushing syndrome; EAS, ectopic adrenocorticotropin secretion; mITT, modified intention to treat; ULN, upper limit of normal.

^a^Percentage and 95% CI not reported if the denominator is less than 10.

^*b*^Based on patients with an available mUFC assessment at baseline.

**Figure 2.**
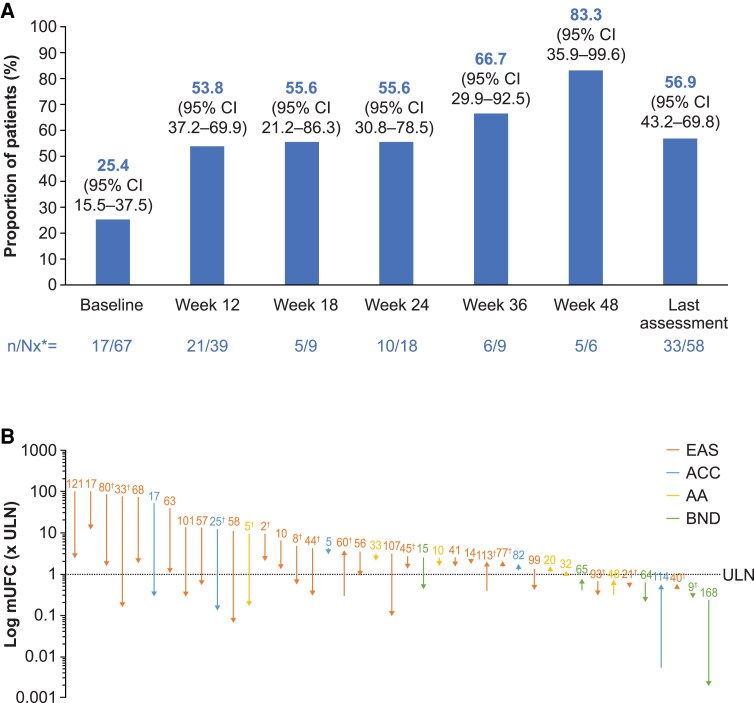
A, Proportion of patients with mean urinary free cortisol (mUFC) less than or equal to the upper limit of normal (ULN) over time in the on-treatment population and B, individual patient values for mUFC at baseline and the last on-treatment assessment. In B, patients with a baseline and at least one on-treatment mUFC measurement were included in this analysis. Numbers represent number of weeks on treatment. Up and down arrows represent an increase or decrease in mUFC from baseline to last assessment. *Nx is the number of patients with evaluable assessments; ^†^Patients received previous medical therapy for Cushing syndrome. Abbreviations: AA, adrenal adenoma; ACC, adrenocortical carcinoma; BND, bilateral adrenal nodular disease; EAS, ectopic adrenocorticotropin secretion.

In the on-treatment population (patients on treatment with an mUFC assessment available at a given time point), the proportion of patients with mUFC less than or equal to the ULN was generally maintained over time; however, data are limited at later time points because of small patient numbers. At last on-treatment assessment, 33 of 58 (56.9%; 95% CI, 43.2%-69.8%) had mUFC less than or equal to the ULN (Fig. 2A). Overall, median (95% CI) time to mUFC normalization was 2.9 months (2.5-6.2 months). See Supplementary Figs. S2 and S3 (26) for the proportion of patients with mUFC less than or equal to the ULN by etiology and type of intervention.

Most patients had a decrease in mUFC from baseline to last assessment. Four patients had a small increase in mUFC from baseline to greater than the ULN at last assessment. Individual patient changes in mUFC from baseline to last on-treatment assessment are shown in Fig. 2B.

In the ITT population, of patients with an available mUFC at weeks 12 and 24, 7 of 45 (15.6%; 95% CI, 6.5%-29.5%) and 6 of 21 (28.6%; 95% CI, 11.3%-52.2%), respectively, had an mUFC greater than the ULN but a 50% or greater reduction from baseline. Outcomes by baseline mUFC severity are shown in Supplementary Table S4 (26).

At week 12, 31 of 48 (64.6%; 95% CI, 49.5%-77.8%) patients in the on-treatment population had ACM less than or equal to the ULN: EAS, n = 15/29 (51.7%; 95% CI, 32.5%-70.6%); ACC, n = 6/7; AA, n = 3/5; BND, n = 7/7. Of patients with available assessments at week 12, those with ACM less than or equal to the ULN by baseline ACM severity were as follows: < 2 × ULN, n = 15/20 (75%; 95% CI, 50.9%-91.3%); 2-5 × ULN, n = 3/7; > 5 × ULN, n = 9/14 (64.3%; 95% CI, 35.1%-87.2%).

**Figure 3.**
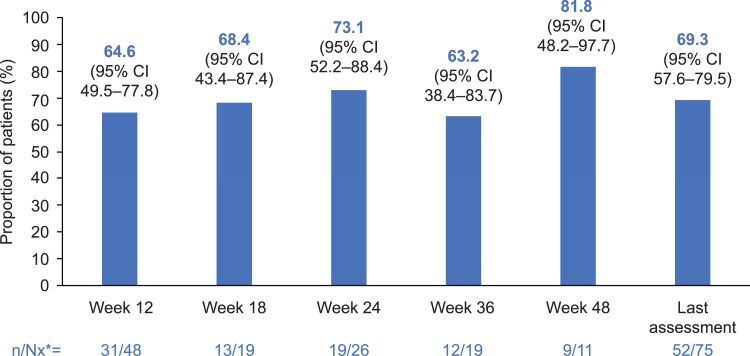
Proportion of patients with any cortisol measure less than or equal to the upper limit of normal over time in the on-treatment population. *Nx is the number of patients with evaluable assessments.

The proportion of patients with ACM less than or equal to the ULN in the on-treatment population was generally maintained over time; however, data are limited at later time points because of small patient numbers. At the last on-treatment assessment, 52 of 75 (69.3%; 95% CI, 57.6%-79.5%) had ACM less than or equal to the ULN (Fig. 3). Overall, median (95% CI) time to ACM normalization was 1.4 months (1.1-2.5 months). See Supplementary Fig. S4 (26) for the proportion of patients with ACM less than or equal to the ULN by etiology. For 14 patients with ACM greater than the ULN at last assessment, most tended to experience reductions from baseline and 8 had ACM less than or equal to the ULN at least once between baseline and their last on-treatment assessment. Individual patient changes in ACM from baseline to last on-treatment assessment are shown in Supplementary Fig. S5 (26).

In the ITT population, of patients with available ACM at weeks 12 and 24, 8 of 55 (14.5%; 95% CI, 6.5%-26.7%) and 4 of 30 (13.3%; 95% CI, 3.8%-30.7%), respectively, had ACM greater than the ULN but a 50% or greater reduction from baseline. Outcomes by baseline ACM severity are presented in Supplementary Table S5 (26).

**Figure 4.**
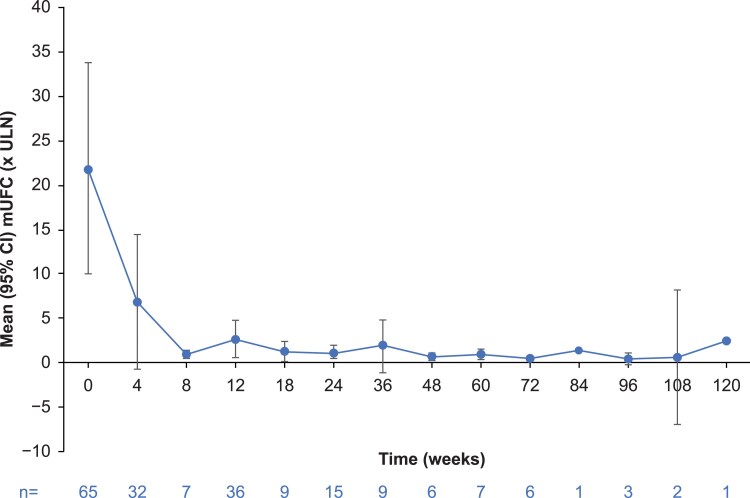
Mean (95% CI) mean urinary free cortisol (mUFC) levels over time in the on-treatment population. No CIs were calculated for weeks 84 and 120. Abbreviation: ULN, upper limit of normal.

Mean mUFC and serum cortisol decreased from baseline to week 4 in the on-treatment population; mUFC then remained stable, whereas serum cortisol showed a further gradual decrease (Fig. 4 and Supplementary Fig. S7A (26)). See the supplementary appendix (26) for further details and outcomes by etiology; data are limited by small patient numbers.

Overall, most cardiovascular and metabolic-related parameters generally remained stable during treatment. See Supplementary Fig. S8 (26) for further details.

### Safety

In total, 87 of 103 (84.5%) patients experienced 1 or more TEAEs: EAS, n = 47/53 (88.7%); ACC, n = 17/19 (89.5%); AA, n = 11/17 (64.7%); BND, n = 12/14 (85.7%). The most common TEAEs (≥10% of patients) were adrenal insufficiency (n = 29/103, 28.2%; investigator assessed), hypokalemia (n = 18/103, 17.5%), asthenia (n = 14/103, 13.6%), diarrhea (n = 13/103, 12.6%) and nausea (n = 12/103, 11.7%; Table 4). TEAEs considered osilodrostat related were reported in 52 of 103 (50.5%) patients, most commonly adrenal insufficiency (n = 26/103, 25.2%; Supplementary Table S6 (26)).

**Table 4.** Overview of treatment-emergent adverse events

	Safety population, N = 103
Any TEAE, n (%)	87 (84.5)
TEAEs reported in ≥5% of patients, n (%)	
Adrenal insufficiency	29 (28.2)
Hypokalemia	18 (17.5)
Asthenia	14 (13.6)
Diarrhea	13 (12.6)
Nausea	12 (11.7)
Acute kidney injury	8 (7.8)
Abdominal pain	7 (6.8)
Anemia	6 (5.8)
Constipation	6 (5.8)
Hypertension	6 (5.8)
Edema peripheral	6 (5.8)
TEAEs leading to dose interruption and/or adjustment, n (%)*^a^*	22 (21.4)
TEAEs or deaths leading to osilodrostat discontinuation, n (%)*^b^*	35 (34.0)

Abbreviation: TEAE, treatment-emergent adverse event.

^*a*^TEAEs leading to dose interruption and/or adjustment (≥2 patients): adrenal insufficiency (n = 8), hypokalemia (n = 3), dehydration (n = 2), nausea (n = 2), vomiting (n = 2).

^*b*^TEAEs leading to osilodrostat discontinuation (≥2 patients): neoplasms benign, malignant, and unspecified (including cysts and polyps; n = 11), death (n = 3), general physical health deterioration (n = 3), cardiorespiratory arrest (n = 2), adrenal insufficiency (n = 2).

Overall, 35 of 103 (34.0%) patients discontinued osilodrostat because of a TEAE or death, most commonly neoplasm progression (n = 11/35, 10.7%; Table 4). Osilodrostat dose was interrupted and/or adjusted in 22 of 103 (21.4%) patients, most commonly for adrenal insufficiency (n = 8/103, 7.8%; Table 4).

Serious TEAEs were reported in 52 of 103 (50.5%) patients: EAS, n = 33/53 (62.3%); ACC, n = 9/19 (47.4%); AA, n = 3/17 (17.6%); BND, n = 7/14 (50.0%); they were most commonly adrenal insufficiency (n = 10/103, 9.7%). Serious TEAEs were considered osilodrostat related in 19 of 103 (18.4%) patients, mostly adrenal insufficiency (n = 10/103, 9.7%; Supplementary Table S7 (26)).

TEAEs considered related to osilodrostat pharmacology were reported in 50 of 103 (48.5%) patients (Table 5). QT prolongation was reported in 2 patients; serum potassium levels did not decrease in these patients, and they did not experience cardiac arrhythmias. Furthermore, osilodrostat dose reduction was not required to manage QT prolongation. Overall, 29 of 103 (28.2%) patients died of a serious TEAE, mostly neoplasm progression (n = 11/103, 10.7%). See the supplementary appendix and Supplementary Tables S8 and S9 (26) for further details.

**Table 5.** Overview of treatment-emergent adverse events potentially related to osilodrostat pharmacology

	Safety population, N = 103	EAS, n = 53	ACC, n = 19	AA, n = 17	BND, n = 14
Any TEAE potentially related to osilodrostat pharmacology, n (%)	50 (48.5)	25 (47.2)	8 (42.1)	6 (35.3)	11 (78.6)
TEAEs by preferred term (≥5% of patients), n (%)^a^					
Related to hypocortisolism					
Adrenal insufficiency	29 (28.2)	16 (30.2)	4 (21.1)	3 (17.6)	6 (42.9)
Related to accumulation of adrenal hormone precursors					
Hypokalemia	18 (17.5)	9 (17.0)	4 (21.1)	2 (11.8)	3 (21.4)
Hypertension	6 (5.8)	3 (5.7)	1 (5.3)	0	2 (14.3)
Edema peripheral	6 (5.8)	2 (3.8)	1 (5.3)	1 (5.9)	2 (14.3)

TEAEs potentially related to osilodrostat pharmacology are those related to hypocortisolism, accumulation of adrenal hormone precursors, QT prolongation, and tumor enlargement.

Abbreviations: AA, adrenal adenoma; ACC, adrenocortical carcinoma; BND, bilateral adrenal nodular disease; EAS, ectopic adrenocorticotropin secretion; TEAE, treatment-emergent adverse event.

^*a*^TEAEs reported in 5% or more of patients in the safety population.

## Discussion

To our knowledge, this is the largest real-world study of steroidogenesis inhibitors solely in patients with nonpituitary CS. This retrospective study of osilodrostat in patients with varying etiologies and severities of hypercortisolism indicates that 44% and 54% of patients in the mITT and on-treatment populations, respectively, had mUFC less than or equal to the ULN at week 12, and 65% in the on-treatment population had ACM (mUFC, morning serum cortisol, LNSC) less than or equal to the ULN at week 12. The proportion of patients with mUFC and ACM less than or equal to the ULN was maintained over time. In the on-treatment population, there was a trend toward a decrease in mean mUFC and serum cortisol over time, although data are limited by small patient numbers. Importantly, most patients had a decrease in mUFC and ACM from baseline to last assessment, regardless of baseline hypercortisolism severity or CS etiology. Cardiovascular and metabolic-related parameters were similar at baseline and last assessment. The osilodrostat safety profile was consistent with that already published (18-22) and the known morbidity of the study population.

Some differences were observed between nonpituitary CS etiologies. The high severity of hypercortisolism and comorbidities in most patients with EAS and ACC, as well as the burden of underlying neoplasms, emphasizes the importance of rapid, effective hypercortisolism control to alleviate cortisol-related comorbidities and improve quality of life (7, 24, 27). Importantly, mUFC decreased by more than 50% in 80% of patients with baseline mUFC greater than 5 × ULN and normalized in 46.7% at week 12. Most patients with baseline mUFC greater than 5 × ULN had EAS or ACC. Aside from UFC, ACM is important to consider given that some patients with severe hypercortisolism were treated according to a block-and-replace strategy, during which the key treatment goal is to block endogenous cortisol production so that levels fall below the lower limit of the normal range (28). Morning serum cortisol prior to hydrocortisone administration is preferred for monitoring patients when using a block-and-replace regimen as UFC may not be a reliable test because exogenous glucocorticoids taken by the patient would be detected in the urine. In patients with baseline ACM greater than 5 × ULN, 64.3% and 26.7%, respectively, had ACM less than or equal to the ULN or a greater than 50% reduction in ACM at week 12. Furthermore, at last assessment, 62.8% of patients with EAS had ACM less than or equal to the ULN and 45.7% had mUFC less than or equal to the ULN. These results are reflective of findings published previously in patients with nonpituitary CS (24) and highlight the benefits of osilodrostat treatment in rapidly normalizing or reducing cortisol levels in patients with severe hypercortisolism. Reducing cortisol levels should lead to fewer or amelioration of hypercortisolism-associated comorbidities (29), as demonstrated in patients with Cushing disease in the LINC 2, 3, and 4 studies (16-21). The effectiveness of osilodrostat has also been demonstrated in a retrospective study of patients with paraneoplastic CS/EAS with severe hypercortisolism (∼10 × ULN), in which 28 of 33 (85%) patients achieved mUFC normalization during osilodrostat treatment (24). In a small case series of 7 patients with ACC and severe hypercortisolism, a significant decrease in UFC and/or serum cortisol was observed in 6 of the 7 patients within 2 weeks of osilodrostat treatment; all patients achieved full control of hypercortisolism at varying time intervals (range, 1 week to 3 months) (25). In our study, patients with EAS and ACC required higher osilodrostat doses to achieve rapid hypercortisolism control, which may also be explained by the higher baseline cortisol levels in these patients.

At baseline, 18 patients had mUFC less than or equal to the ULN; 10 patients, including 6 with EAS, had received previous medical therapy for CS. Some patients, mostly those with BND, had mild disease at baseline.

Hypertension and diabetes mellitus are common comorbidities in patients with CS, present in approximately 80% and 40% to 45% of patients, respectively, at diagnosis, which contributes to the increased morbidity and mortality risk observed in these patients (30-32). Most cardiovascular and metabolic-related parameters were similar at baseline and last assessment. However, precise retrospective analysis of clinical conditions is limited by the multiple parameters involved in blood pressure and glycemic control, including nutritional status, inflammatory and infectious status, burden of the underlying neoplasm, and antihypertensive and antidiabetic medication changes; all of these parameters provide important information but could not be collected in this retrospective study. Robust prospective studies have precisely analyzed clinical end points (16-21), and few retrospective studies have shown that osilodrostat improves comorbidities following cortisol reduction in patients with CS (24, 25).

Of 29 patients who died of a serious TEAE, most (n = 24) had EAS; 5 had ACC. Notably, only one patient with EAS was eligible for surgery. The high death rate observed in these patients may be explained by the increased mortality over other etiologies of CS (7, 27, 29), demonstrated by a median 5-year overall survival of 15% to 70% (7, 9, 33). In patients with EAS and ACC, osilodrostat is effective and well tolerated, with few drug-drug interactions and a lower pill burden than other cortisol-lowering drugs (28, 34, 35). Controlling hypercortisolism in patients with underlying neoplasms can reduce morbidity and mortality associated with hypercortisolism and improve quality of life (7, 8, 27). Findings from this study are consistent with data reported for osilodrostat in patients with Cushing disease (16-21) and nonpituitary CS (24).

Some AEs, including hypocortisolism and hypokalemia, are expected based on the mechanism of action of steroidogenesis inhibitors, including osilodrostat (28). Osilodrostat may result in an increase in adrenal hormone precursors, which can lead to hypokalemia (36). Hypokalemia was one of the most common AEs reported (17.5% of patients). A similar prevalence of hypokalemia has been described in a prospective, interventional study of osilodrostat, in which 13.1% of patients experienced this AE (18, 21). Importantly, the timing of hypokalemia could not be characterized in our study. Hypokalemia is a common comorbidity in patients with severe hypercortisolism and has been shown to improve following cortisol reduction during treatment with steroidogenesis inhibitors (24, 35).

Hypokalemia is a common risk factor for QT-interval prolongation; therefore, it is important to correct hypokalemia before osilodrostat initiation and monitor patients regularly during treatment (36). However, AEs related to QT prolongation were infrequent in this real-world study.

Adrenal insufficiency was reported in 28.2% of patients. Patients with BND may be at increased risk of hypocortisolism-related events potentially because cortisol levels tend to be normal or mildly elevated before treatment initiation. Factors that can increase the risk of adrenal insufficiency were not analyzed in our study. However, based on the published literature, patients with EAS and ACC are prone to infections (37, 38), which can precipitate adrenal insufficiency (39, 40). As such, specialist care is required for this subset of patients. In addition, some authors have attributed the potential for adrenal insufficiency during osilodrostat treatment to the efficacy of the drug, a favorable characteristic given the prognosis of patients with uncontrolled CS (24). Adrenal insufficiency may reflect both drug potency and limited experience of using osilodrostat for the treatment of adrenal CS at the time of the study. It can be expected that, with increased clinical experience and a better understanding of the osilodrostat safety profile, the prevalence of adrenal insufficiency (the most common TEAE in this study) may decrease in the future. Accordingly, patients should be monitored regularly and educated on the symptoms of adrenal insufficiency to allow for prompt management (40, 41). Notably, few patients discontinued osilodrostat because of TEAEs, including those with BND, suggesting that events were manageable. Although data on the management of these events were not available, data from the osilodrostat clinical trial program in patients with CS have demonstrated that hypocortisolism-related events are mostly manageable with temporary dose interruption/adjustment and/or glucocorticoid therapy without discontinuation (16-22). As this was a retrospective study, cortisol data were generally not available at the time of an event. As such, further analyses are required to understand whether events were true chronic or acute adrenal insufficiency vs glucocorticoid withdrawal syndrome. These findings are similar to those reported in another retrospective study conducted in patients with paraneoplastic CS/EAS, in which adrenal insufficiency was reported in 8 of 33 (24%) patients (24).

One of the merits of this study lies in the large patient population with nonpituitary CS that was enrolled, which will provide further evidence to support the use of steroidogenesis inhibitors in patients with nonpituitary CS given the limited number of studies that have been published previously (1, 2).

This retrospective study had some limitations. Data were collected through review of individual patient medical records, which had missing information, leading to small patient numbers at several time points, particularly during long-term treatment. As the date of the final osilodrostat dose was missing for some patients, osilodrostat exposure data are based on end-of-treatment dates estimated using a conservative approach. There were fewer available assessments of cortisol and other biochemical parameters, and less safety data, than in prospective interventional studies. Some patients had only single UFC measurements at certain visits, which was classed as their mUFC for that visit; guidelines recommend that the mean of 2 or more measurements be recorded (42, 43). There were also difficulties with measuring UFC in some patients because of urinary incontinence, transient or permanent renal insufficiency, and emergency clinical situations, especially in patients with EAS. In these situations, patients were treated according to titration and block-and-replace regimens. When monitoring patients with severe hypercortisolism, serum cortisol measurements are used rather than UFC, as discussed earlier. This emphasizes the importance of considering ACM in patients with severe hypercortisolism (28). Data for AEs related to accumulation of adrenal hormone precursors may not always have been collected by the investigator for the following potential reasons: focus on rapidly normalizing cortisol levels in patients with life-threatening hypercortisolism, difficulty analyzing hyperandrogenism in female patients with ACC as these tumors may be associated with androgen excess, and lack of data collection during a short time period for patients receiving short-term osilodrostat in preparation for surgery. However, these reasons are speculative, and further investigation would be needed to determine the exact reason for the lower occurrence of AEs related to accumulation of adrenal hormone precursors than reported in previous LINC studies. Prescribing patterns were not controlled; the initial osilodrostat dose and dose titration were highly variable. Limited experience with the drug in some situations may have influenced the proportion of patients achieving normal cortisol levels by week 12. Increasing clinical experience may in part explain the increased number of patients with cortisol control over time. Future analyses evaluating the effectiveness and safety of osilodrostat by type of intervention would be of interest. The clinical benefit of osilodrostat on comorbidities related to CS could not be specified. Retrospective analysis of cardiovascular and metabolic-related parameters, such as blood pressure and glycemic parameters, was limited by small patient numbers at most time points; detailed data on concomitant medications for managing comorbidities are not available. However, data from the osilodrostat clinical development program and studies of other medical therapies have demonstrated an improvement in clinical features and quality of life following a decrease in cortisol production in patients with CS (16-21, 24, 44-46).

## Conclusions

This study describes real-world osilodrostat use in patients with varying etiologies and severities of hypercortisolism. Data show that osilodrostat normalized or considerably reduced cortisol levels regardless of baseline cortisol level in patients with nonpituitary CS, including those with severe hypercortisolism and underlying neoplasms. Safety events are consistent with the established safety and efficacy profile reported in patients with Cushing disease (16-21) and nonpituitary CS (22). These data affirm that patients should be monitored regularly and educated on the safety profile of osilodrostat and the signs and symptoms of adrenal insufficiency to minimize the occurrence of these AEs. Overall, this real-world study shows that osilodrostat is a suitable treatment for endogenous CS of various nonpituitary etiologies.

## Data Availability

The datasets generated and analyzed during the current study are not publicly available but are available from the corresponding author on reasonable request. Recordati Rare Diseases will share the complete deidentified patient dataset, study protocol, statistical analysis plan, and informed consent form on request, effective immediately following publication, with no end date.

## Clinical Trial Information

ClinicalTrials.gov registration number NCT05633953 (registered December 1, 2022).

## Abbreviations

AA: adrenal adenoma
ACC: adrenocortical carcinoma
ACM: any cortisol measure
AE: adverse event
BND: bilateral adrenal nodular disease
CS: Cushing syndrome
EAS: ectopic adrenocorticotropin secretion
ITT: intention to treat
LNSC: late-night salivary cortisol
mITT: modified intention to treat
mUFC: mean urinary free cortisol
TEAE: treatment-emergent adverse event
ULN: upper limit of normal
